# Rosiglitazone treatment restores renal responsiveness to atrial natriuretic peptide in rats with congestive heart failure

**DOI:** 10.1111/jcmm.14366

**Published:** 2019-05-13

**Authors:** Ilia Goltsman, Emad E. Khoury, Doron Aronson, Omri Nativ, Giora Z. Feuerstein, Joseph Winaver, Zaid Abassi

**Affiliations:** ^1^ Department of Physiology Bruce Rappaport Faculty of Medicine Technion‐IIT Haifa Israel; ^2^ Department of Cardiology Rambam Health Care Campus Haifa Israel; ^3^ FARMACON LLC, Translational Medicine Company Bryn Mawr Pennsylvania; ^4^ Department of Laboratory Medicine Rambam Health Care Campus Haifa Israel

**Keywords:** ANP, heart failure, kidney excretion, rat, thiazolidinedione

## Abstract

The thiazolidinedione (TZD) class of Peroxisome proliferator‐activated receptor gamma agonists has restricted clinical use for diabetes mellitus due to fluid retention and potential cardiovascular risks. These side effects are attributed in part to direct salt‐retaining effect of TZDs at the renal collecting duct. A recent study from our group revealed that prolonged rosiglitazone (RGZ) treatment caused no Na+/H_2_O retention or up‐regulation of Na^+^ transport‐linked channels/transporters in experimental congestive heart failure (CHF) induced by surgical aorto‐caval fistula (ACF). The present study examines the effects of RGZ on renal and cardiac responses to atrial natriuretic peptide (ANP), Acetylcholine (Ach) and S‐Nitroso‐*N*‐acetylpenicillamine (SNAP‐NO donor). Furthermore, we assessed the impact of RGZ on gene expression related to the ANP signalling pathway in animals with ACF. Rats subjected to ACF (or sham) were treated with either RGZ (30 mg/kg/day) or vehicle for 4 weeks. Cardiac chambers pressures and volumes were assessed invasively via Miller catheter. Kidney excretory and renal hemodynamic in response to ANP, Ach and SNAP were examined. Renal clearance along with cyclic guanosine monophosphate (cGMP), gene expression of renal CHF‐related genes and ANP signalling in the kidney were determined. RGZ‐treated CHF rats exhibited significant improvement in the natriuretic responses to ANP infusion. This ‘sensitization’ to ANP was not associated with increases in neither urinary cGMP nor in vitro cGMP production. However, RGZ caused down‐regulation of several genes in the renal cortex (Ace, Nos3 and Npr1) and up‐regulation of ACE2, Agtrla, Mme and Cftr along down‐regulation of Avpr2, Npr1,2, Nos3 and Pde3 in the medulla. In conclusion, CHF+RGZ rats exhibited significant enhancement in the natriuretic responses to ANP infusion, which are known to be blunted in CHF. This ‘sensitization’ to ANP is independent of cGMP signalling, yet may involve post‐cGMP signalling target genes such as ACE2, CFTR and V2 receptor. The possibility that TZD treatment in uncomplicated CHF may be less detrimental than thought before deserves additional investigations.

## 1 INTRODUCTION

Congestive heart failure and type 2 diabetes mellitus (T2DM) are leading causes of morbidity and mortality in developed countries.^1^, ^2^ Therefore, due to the substantial burden of these two clinical conditions confounded by their combination, pharmacotherapy for DM faces the challenges of successfully managing the disease considering the accompanying restrictions imposed by CHF and of modulating the cardiovascular risk inherent to T2DM.^3^ Originally, thiazolidinediones (TZDs), especially the synthetic full agonists of proliferator‐activated receptor gamma (PPARγ), have been acknowledged for their beneficial impact in management of T2DM.^4^ However, over time and following robust and prolonged clinical experience, issue arose regarding risk benefits of this class of drugs. In particular, T2DM confounded by heart failure poses significant limitation for the use of TZDs due to increased risk of cardiac ischaemic events, peripheral oedema and fluid retention, especially in patients with CHF.^5^, ^6^, ^7^, ^8^, ^9^ Indeed, a major hallmark of CHF, fluid retention, contributes to the major debilitating symptoms of the disease.^10^ It is largely attributed to a combination of mechanisms: (a) altered renal haemodynamics, as manifested by compromised glomerular filtration rate (GFR) and renal blood flow (RBF); (b) activation of sodium‐retaining neurohormonal systems; and (c) attenuation of the vascular function of natriuretic systems. Both low‐ and high‐cardiac output states are associated with reduced renal perfusion pressure and tubular sodium delivery.^11^, ^12^ Hence, compensatory neurohormonal and local renal effectors are triggered to preserve the effective circulating volume and maintain perfusion to vital organs.^10^, ^11^, ^12^ These compensatory responses include various neurohormonal vasoconstrictor systems such as the renin‐angiotensin‐aldosterone system (RAAS), sympathetic nervous system, arginine vasopressin (AVP) and endothelin‐1 (ET‐1), all known potent vasoconstrictor and salt‐conserving hormones.^11^, ^12^, ^13^, ^14^ Fluid retention in both clinical and experimental CHF is associated with elevated atrial natriuretic peptide (ANP) and brain natriuretic peptide (BNP), indicating a blunted renal response to these natriuretic peptides (NPs).^10^, ^15^, ^16^, ^17^, ^18^, ^19^ Evidence suggests that the mechanism for TZD‐induced fluid retention involves primarily direct salt‐retaining effects of PPAR‐γ agonists on the nephron via up‐regulation of epithelial sodium channel (ENaC) in the collecting duct.^20^ However, a recent study from our laboratory concluded that chronic exposure to RGZ in a rat model of volume overload had actually improved renal and cardiac response to volume overload.^21^ The aim of the present study was to explore potential mechanism(s) that may underlie RGZ adverse effects in CHF.

## 2 MATERIALS AND METHODS

Studies were performed on male Sprague‐Dawley rats, weighing ~300 g. All animal experiments were approved and performed according to the Guide for the Care and Use of Laboratory Animals (NIH Publication No. 85‐23, revised 1996) and approved by the committee for the supervision of animal experiments, Technion, IIT.

### 2.1 Experimental model

An aorto‐caval fistula (ACF) was surgically created by introducing a fistula between the abdominal aorta and the inferior vena cava distal to the origin of the renal arteries.^22^, ^23^ After recovery, each single rat was placed in a metabolic cage and monitored for urine sodium excretion. Based on the daily Na^+^ excretion (UNa$\dot{V}$) over 7 days, only rats with ‘compensated’ CHF (UNaV >1200 μEq/day) were used for further studies, to avoid the confounding effects of pre‐existing salt and water retention in ‘decompensated’ condition.

#### 2.1.1 Experimental groups

The conducted studies included the following experimental groups:

1. Sham‐operated control rats, treated with vehicle, a drug‐free buffer (Control+Veh).
2. Sham‐operated control rats, treated with RGZ (Control+RGZ).
3. Rats with ACF‐compensated, treated with vehicle (CHF+Veh).
4. Rats with ACF‐compensated, treated with RGZ (CHF+RGZ).

#### 2.1.2 Chronic RGZ treatment

Rats were housed for baseline assessment over 5 days. ACF or sham operation followed and maintained for five additional weeks. Seven days after the operation, rats with compensated CHF were started on either RGZ treatment (30 mg/kg/day, dissolved in 1 mL of vehicle solution consisting of: 0.4 mL 5% Tween‐80, 0.25 mL 2% methyl cellulose and 0.35 mL double distilled water) or the vehicle only (1 mL/day) by oral gavage for 4 weeks. A matched control group of sham‐operated rats was similarly treated with either RGZ or vehicle. After 4 weeks of treatment, haemodynamic and genomic studies were performed:

### 2.2 Haemodynamic studies

#### 2.2.1 Renal response to ANP infusion

The effects of RGZ treatment on renal function and haemodynamic response to ANP infusion were evaluated by clearance methodology in rat with ACF or sham operation (n = 8‐10). Rats were anaesthetized with Inactin (100 mg/kg BW), placed on a thermoregulated surgical table and prepared for clearance studies as described previously.^23^ Briefly, following tracheotomy, the left carotid artery was catheterized and MAP was recorded and the right jugular vein was catheterized for fluid infusion. A catheter was inserted via cystostomy for urine collection. A low dose (15 μg/kg/min) and high dose (50 μg/kg/min) of ANP were dissolved in saline, preceded by a prime bolus injection of the same dose, maintained for 1 hour. Urine and plasma samples were collected in vials and stored in −20°C.

#### 2.2.2 Renal haemodynamic responses to endothelium‐dependent and endothelium‐independent vasodilators

In separate groups of rats, the effects of RGZ treatment on the renal haemodynamic responses to the endothelium‐dependent vasodilator, Ach (N = 7‐10) and the endothelium independent NO‐donor, *SNAP* (N = 7‐8) were evaluated. Following inactin anaesthesia, rats were prepared as described above. RBF was measured using an ultrasonic flow probe (type 1RB) connected to an ultrasonic flowmeter (model T206, Transonic Corp Inc., Ithaca, NY, USA), as previously described.^24^, ^25^ RBF and mean arterial blood pressure (MAP) were continuously recorded by a computerized data acquisition system. Renal vascular resistance (RVR) was calculated by the standard formula (RVR = MAP/RBF) and expressed as resistance units (RU). ACh (Sigma‐Aldrich) was infused intravenously in incremental doses (1, 10, 100 μg/kg/min) over a 30‐min period for each dose and followed by a 30‐45 min recovery period. SNAP was infused in two doses: 10 and 30 μg/kg over a 30 min for each dose and followed by a 30‐min recovery period.

Measurements of renal clearance parameters and UcGMP excretion were also performed in response to a representative dose of ACh (10 μg/kg/min, in which maximal renal vasodilatation was observed) in separate groups of rats (N = 5 each). The preparations were made as described for the ANP response protocol above, and experiments consisted of baseline, ACh infusion and recovery periods of 30‐40 min each.

#### 2.2.3 Cardiac function

Cardiac function was monitored by inserting a Millar cardiac conductance catheter (Mikro‐Tip^®^; Millar Instruments, Houston, TX, USA) to the left ventricle (LV) via the carotid artery. LV pressures, volumes and derivations of cardiac pressure‐volume relationships were continuously measured.

### 2.3 Biochemical analysis

#### 2.3.1 cGMP production in response to ANP

Cyclic guanosine monophosphate production capacity was determined in glomeruli and collecting ducts isolated from kidneys of the same experimental groups in response to ANP infusion (N = 5‐7) as described previously.^26^, ^27^ Urinary and tissue cGMP concentrations were measured through a commercially available ELISA kit (Rat cGMP EIA Kit; Cayman Chemical, Ann Arbor, MI, USA) and protein concentrations using the Bio‐Rad Protein Assay reagent (Bio‐Rad, Hercules, CA, USA).^28^

#### 2.3.2 Gene expression of signal transduction

In order to associate the physiological and pharmacological studies with genomic alterations, the following RNA transcripts were monitored: natriuretic peptide receptors and metabolizing enzymes, intracellular cGMP synthetic pathways elements, cGMP‐metabolizing phosphodiesterases and other regulators of sodium and water transport. Glomeruli and collecting ducts were isolated as described above. Total RNA was prepared and cleaned using the RNAqueous^®^‐4PCR kit (Ambion, Austin, TX, USA). Following RNA and cDNA preparation, quantitative real‐time PCR was performed utilizing custom‐designed TaqMan^®^ Low Density Arrays (TLDA) from Applied Biosystems as described previously.^29^ The comparative C_T_ method of relative quantification was used for data analysis.^30^ Averaged values for GUSB, PPIA and GAPDH used as normalizers, compared to the C_T_ value of the target gene (ΔC_T_). Relative quantification (RQ or fold change) between different sample groups was then determined according to the $2^{-\Delta \Delta {\text{C}}_{T}}$ method as described above. The mean of the expression values for the control + vehicle samples (N = 3) was used as the calibrator for these calculations.

Of the 32 examined genes, 19 were selected for a validation study. Inclusion criteria were either a trend of altered gene expression in the pilot study (expression difference of at least 30%‐40% compared with the control group with a *P* < 0.15) or strong physiologic relevance based on current literature. The comparative C_T_ method of relative quantification was used. Either averaged values for glucuronidase beta (GUSB), PPIA and GAPDH for medullary tissues or the housekeeper among those which were closest in expression range to the target for cortical tissues, served as normalizers. Relative quantification (RQ or fold change) between different sample groups was then determined according to the 2^−ΔΔCT^ method as described above. The mean of the expression values for the control + vehicle samples (N = 5‐6) was used as the calibrator for these calculations.

### 2.4 Renal function analysis

Urine and plasma electrolytes were measured by flame photometer (model 943; Instrumentation Laboratory, Milano, Italy). Inulin concentrations were determined using the anthrone method.^31^

### 2.5 Statistical analysis

One‐way analysis of variance (ANOVA) and two‐way ANOVA for repeated measurements were used for group comparison, as appropriate. Tukey's and Bonferroni's corrections for multiple comparisons were used as ANOVA post hoc tests respectively. Repeated measures one‐way ANOVA, followed by Dunnett's multiple comparison test, was used to test significance of change from baseline values of clearance parameters within treatment groups in the volume expansion experiments. *P* = 0.05 was chosen as the significance level for all analyses. Data are expressed as means ± SEM.

## 3 RESULTS

### 3.1 Effects of RGZ treatment on renal natriuretic and haemodynamic responses to ANP infusion

**Figure 1:**
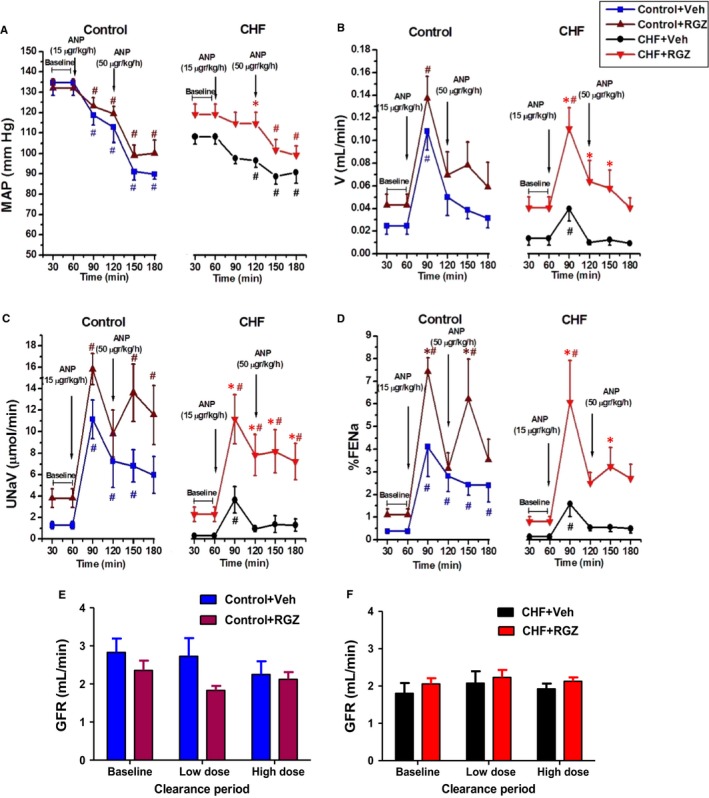
Effects of RGZ treatment on renal excretory and haemodynamic parameters in response to ANP infusion. A, MAP, (B) V, (C) U_N__a_V, (D) FE_N__a_ and (E, F) GFR during clearance experiments in control rats and CHF animals. Low and high doses designate 15 and 50 μg/kg/min of ANP respectively. The baseline period values were calculated as the average of urine collections at 30 and 60 min; the low‐dose period values were calculated as the average of urine collections at 90 and 120 min; the high‐dose period values were calculated as the average of urine collections at 150 and 180 min. Data represent the mean ± SEM. N = 8‐10 for all groups. *P < 0.05 vs Veh‐treated counterparts; ^#^ *P* < 0.05 vs baseline period (up to 60 min)

The natriuretic/diuretic response to ANP infusion and related changes in GFR and MAP in control and CHF rats treated with either RGZ or Veh are shown in Figure 1. In all four groups, continuous infusion of both low (15 μg/kg/min) and high dose (50 μg/kg/min) of ANP following a priming bolus resulted in significant increases in V, U_Na_V and FE_Na_ that were offset to a certain degree by a parallel decrease in MAP (Figure 1A‐D). The increases in U_Na_V, V and FE_Na_ in response to both ANP doses in Veh‐treated CHF rats were significantly smaller than those observed in Veh‐treated control rats (Figure 1A‐D). RGZ treatment restored the natriuretic and diuretic responses in CHF rats compared with their Veh‐treated counterparts, as seen in Figure 1A‐D. Notably, the baseline excretory parameters in both CHF and control rats treated with RGZ tended to be higher than Veh‐control counterparts (*P* = NS). This trend persisted even after comparing the fold increase among the investigated groups.

As shown in Figure 1E and F, basal GFR was lower in CHF rats as compared with Control+Veh. Yet, the difference did not reach statistical significance. Interestingly, RGZ tended to increase MAP in CHF rats compared with Veh‐treated counterparts during the entire experimental course (*P* < 0.05 for MAP difference at 90‐120 min); however, it remained much less than in control animals (Figure 1A).

**Figure 2:**
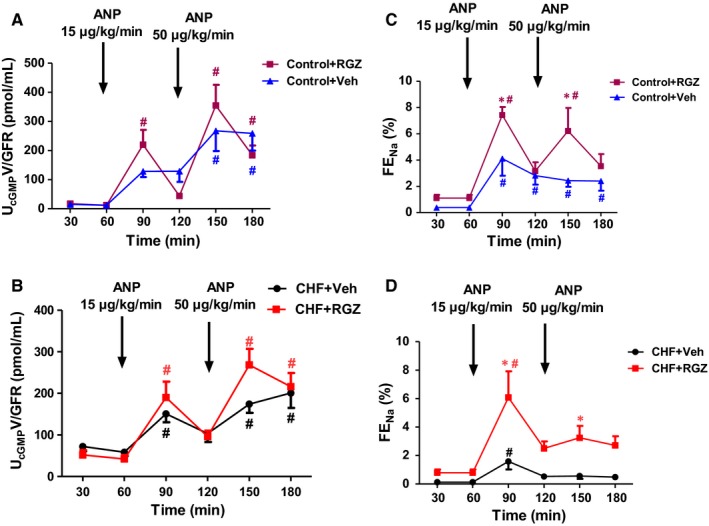
Effects RGZ treatment on U_c_ _GMP_ excretion rate in response to ANP infusion. U_c_ _GMP_V normalized to GFR (U_c_ _GMP_V/GFR) in response to ANP infusion in control (A) and CHF rats (B) (upper and lower right), compared with their respective FE_N__a_ levels (upper, C and lower left, D, respectively, taken from Figure 1). Data represent the mean ± SEM (N = 8‐10). *P < 0.05 vs Veh‐treated counterparts; ^#^ *P* < 0.05 vs baseline period (up to 60 min)

Figure 2A and B presents the urinary cGMP excretion rates (U_cGMP_V, normalized to GFR) in response to ANP infusion compared with the FE_Na_ graphs seen in Figure 1. As expected, U_cGMP_V increased following ANP administration in both doses into treated and untreated CHF rats and their controls (Figure 2A and B). However, the treatment with RGZ did not influence the U_cGMP_V in response to ANP in both subgroups.

### 3.2 Effects of RGZ treatment on renal natriuretic and vasodilatory responses to ACh infusion

**Figure 3:**
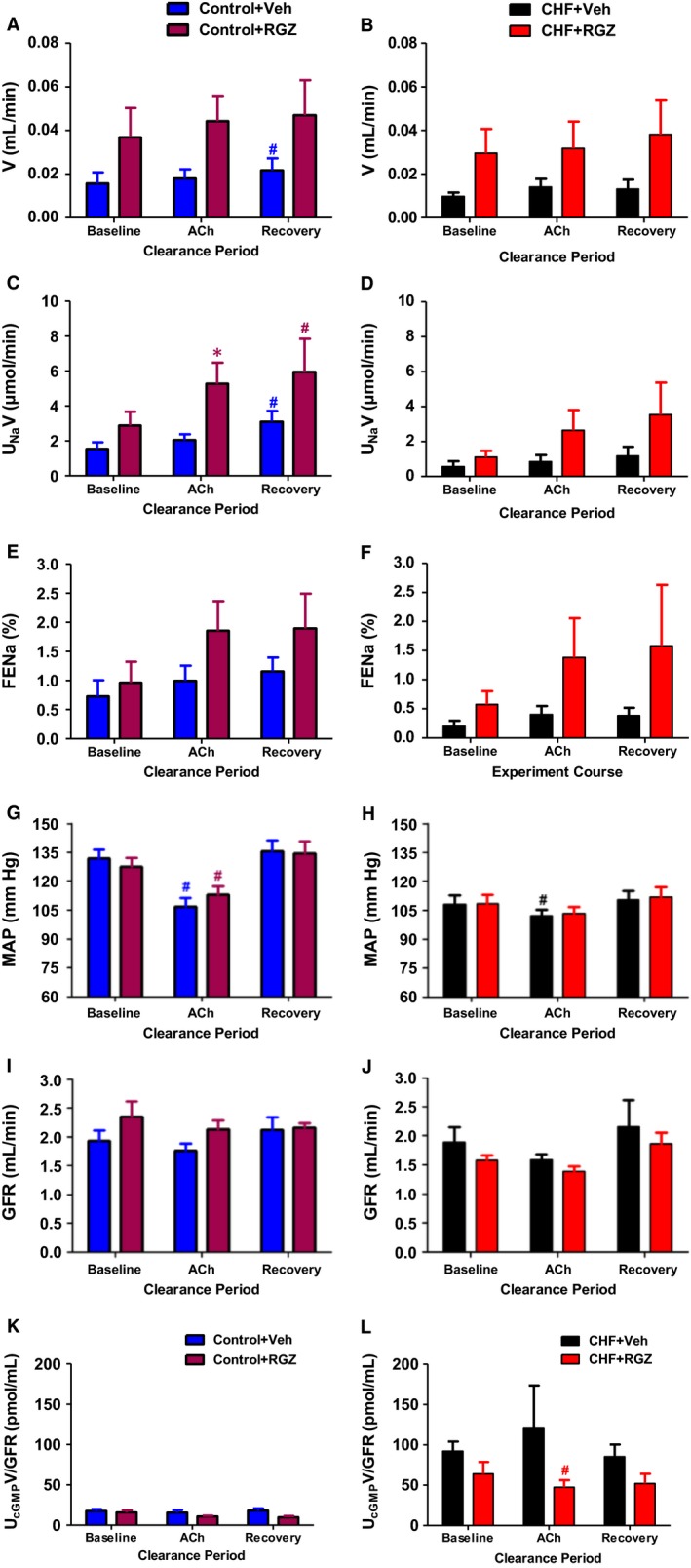
Effects of RGZ treatment on renal excretory and haemodynamic parameters in response to ACh infusion. A, V, (C) U_N__a_V and (E) FE_N__a_, (G) MAP, (I) GFR, (K) U_c_ _GMP_V normalized to GFR (U_c_ _GMP_V/GFR) during clearance experiments in control group; (B) V, (D) U_N__a_V and (F) FE_N__a_, (H) MAP, (J) GFR, (L) U_c_ _GMP_V normalized to GFR (U_c_ _GMP_V/GFR) during clearance experiments in CHF rats. Each clearance period lasted 30‐40 min. *P < 0.05 vs Veh‐treated counterparts; ^#^ *P* < 0.05 vs baseline period. ^#^ *P* < 0.05 vs baseline period . Data represent the mean ± SEM of N=7‐10 in each group

The natriuretic/diuretic response to ACh infusion and related changes in GFR and MAP in control and CHF rats treated with either RGZ or Veh is shown in Figure 3. Administration of ACh (10 μg/kg/min) induced negligible increases from baseline in V, U_Na_V and FE_Na_ during the infusion and recovery (after termination of ACh infusion) periods in both control and CHF groups (Figure 3A‐F). Statistically significant increases from baseline were confined to U_Na_V in both control groups and V in the control+Veh group during the recovery period (*P* < 0.05) (Figure 3C). Simultaneously, MAP decreased significantly during ACh infusion and returned to normal levels during the recovery phase (Figure 3G and H). No significant increases from baseline in V, U_Na_V and FE_Na_ were seen in response to ACh in both CHF subgroups (Figure 3B, D and F).

In this study, RGZ treatment tended to increase natriuretic and diuretic responses to ACh infusion in both CHF and control rats compared with their Veh‐treated counterparts, yet only U_Na_V was found to be significant only in control+RGZ rats during the infusion period (*P* < 0.05 vs control+Veh) (Figure 3C). Figure 3K and L compares the U_cGMP_V normalized to GFR following ACh stimulation. Basal levels of cGMP were significantly higher in the CHF group compared to the control group. Compared to the corresponding pattern of U_cGMP_ excretion seen in response to ANP administration, there was no significant increase from baseline in U_cGMP_V in response to Ach administration. Moreover, RGZ treatment did not increase the urinary excretion of cGMP and in the CHF group rather it caused a decrease in cGMP excretion following Ach infusion.

### 3.3 Renal haemodynamic responses to SNAP: endothelium‐independent vasodilator

**Figure 4:**
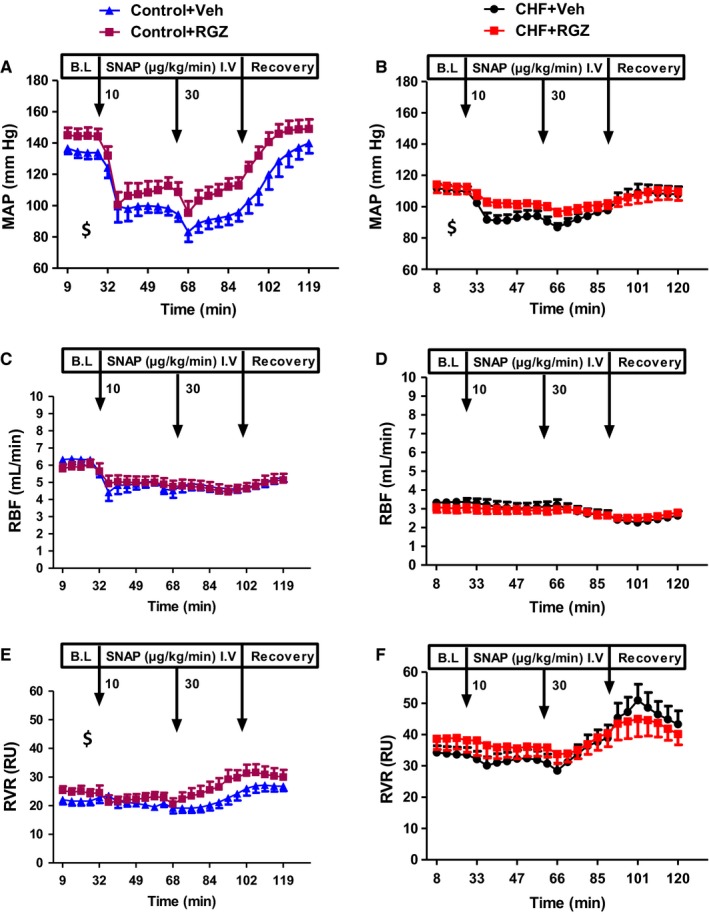
Effects of RGZ treatment on the renal vasodilatory response to SNAP infusion. A, MAP, (C) RBF and (E) RVR during clearance experiments in control group; (B) MAP, (D) RBF and (F) RVR during clearance experiments in CHF rats. ^$^ *P* < 0.05 for the comparison of the curve representing RGZ treatment with that representing Veh treatment by two‐way ANOVA for repeated measurements (significant treatment effect), however without significant differences at specific time points in the post hoc analysis. No difference was observed in RBF between the CHF groups. Data represent the mean ± SEM of N = 7‐8 rats in each group

Figure 4 summarizes the renal and systemic haemodynamic responses to sequentially increasing doses of SNAP (endothelium‐independent vasodilator) in all four groups. SNAP infusion in control rats elicited marked and dose‐dependent systemic vasodilation, while maximal RBF response was observed with the lower SNAP dose before being offset by further decrease in MAP or other physiological compensatory mechanisms Figure 4A, C and E). These responses were significantly attenuated in the CHF group (Figure 4B, D and F). No significant changes in MAP in RGZ‐treated control or CHF rats were noted during the entire experimental period as compared with their Veh‐treated counterparts. In addition, no difference in RBF was seen between the RGZ‐ and Veh‐treated groups, CHF or control, during the entire experimental period.

### 3.4 Cardiovascular haemodynamics and mechanics

**Figure 5:**
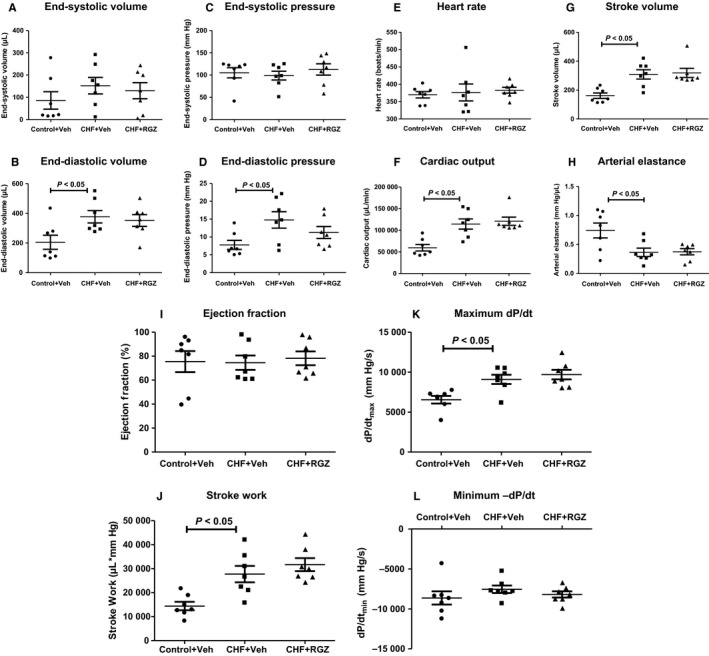
Effects of chronic RGZ treatment on LV pressures and volumes. A, End‐systolic volume, (B) end‐diastolic volume, (C) end‐systolic pressure, (D) end‐diastolic pressure, (E) heart rate, (F) cardiac output, (G) stroke volume, (H) arterial elastance, (I) ejection fraction, (J) stroke work, (K) maximal dP/dt and (L) minimal dP/dt. Ejection fraction equals (end‐diastolic volume – end‐systolic volume)/end‐diastolic volume. Stroke work is calculated as the area under the pressure‐volume curve (volume*pressure product). Maximum and minimum dP/dt signify the maximal systolic pressure generation as a parameter of LV contractility and the minimal diastolic pressure loss as a parameter of LV relaxation capacity respectively. Data represent mean ± SEM of N = 7 in each group. *P* < 0.05 vs Control+Veh

After 4 weeks following ACF, the hearts of operated rats function in a compensated manner with regard to pressure generation as demonstrated in Figure 5. CHF+Veh rats exhibited significantly increased end‐diastolic volume, as already demonstrated by their pressure‐volume relationship, compared with control+Veh rats (*P* < 0.05) (Figure 5B). Untreated CHF rats also had increased end‐diastolic pressure, a parameter of LV wall stress (*P* < 0.05) (Figure 5D).

Congestive heart failure rats displayed significantly increased stroke volume and cardiac output compared with controls (*P* < 0.05 for both) (Figure 5G and F) and significantly decreased arterial elastance (a measure of effective arterial afterload, *P* < 0.05) (Figure 5H), while having the same heart rate (Figure 5E). RGZ treatment in CHF rats did not modify any of these parameters. LV ejection fraction was unaltered in CHF rats compared with controls and it was also not affected by RGZ treatment in CHF rats (Figure 5I). Also, the systolic parameters of stroke work and maximal pressure gradient during systole were increased in CHF rats compared with controls (*P* < 0.05 for both) (Figure 5K and L), but were not affected following RGZ treatment.

### 3.5 cGMP generation

**Figure 6:**
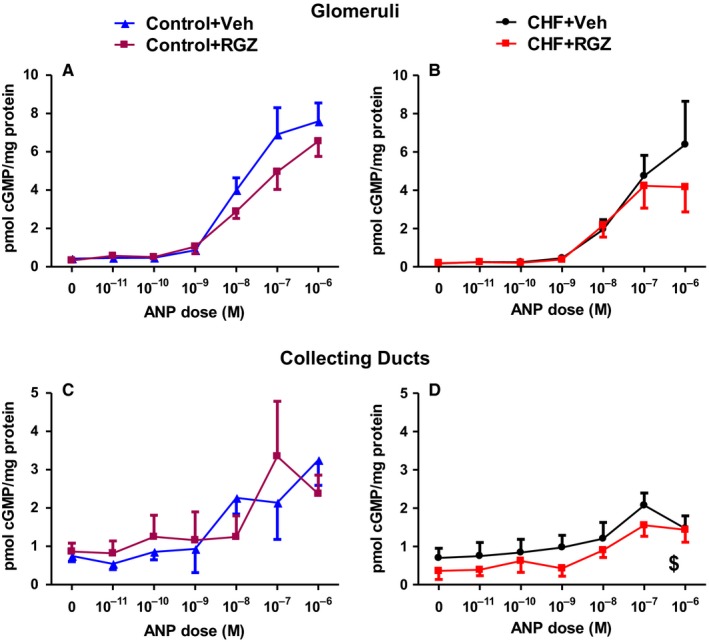
Effects of RGZ treatment on renal cGMP generation in response to ANP in vitro. cGMP production normalized to tissue sample protein concentration in response to ANP in glomeruli (A, B) and inner medulla (C, D) in CHF (B,D) and control (A, C) rats. Data represent the mean ± SEM of N = 5‐7 rats in each tissue type and group. ^$^ *P* < 0.05 for the comparison of the curve representing RGZ treatment with that representing Veh treatment by two‐way ANOVA for repeated measurements (significant treatment effect), however without significant differences at specific time points in the post hoc analysis

The effect of RGZ treatment on renal cGMP generation at the glomeruli and collecting ducts was measured in vitro in all four groups. As shown in Figure 6, RGZ treatment neither changed cGMP response in CHF nor control rats in any of the tissues examined.

### 3.6 Effects of RGZ treatment on gene expression related to ANP signalling

Noticing a favourable effect of RGZ treatment on the renal tubular action of ANP in CHF rats, but not sham‐operated animals, we hypothesized that an interaction may occur between PPARγ and the gene expression level of the renal ANP system. Thus, following a literature review to find putative RGZ‐regulated genes related to ANP signalling, a screening study was conducted to test whether RGZ treatment alters specific gene expression in CHF rats (Table 1).

**Table 1:** Probes used for validation of PPARgamma‐regulated gene targets

Gene symbol	Gene name	Assay ID (Applied Biosystems)
Ace	Angiotensin I converting enzyme	Rn00561094_m1
Ace2	Angiotensin I converting enzyme 2	Rn01416293_m1
Agtr1a	Angiotensin II receptor, type 1a	Rn02758772_s1
Avpr2	Arginine vasopressin receptor 2	Rn00569508_g1
Cftr	Cystic fibrosis transmembrane conductance regulator	Rn01455979_m1
Corin	Corin (Serine peptidase, atrial natriuretic peptide‐converting enzyme)	Rn00711040_m1
Gapdh	Glyceraldehyde‐3‐phosphate dehydrogenase	Rn01775763_g1
Gusb	Glucuronidase, beta	Rn00566655_m1
Mas1	MAS1 oncogene	Rn00562673_s1
Mme	Membrane metalloendopeptidase	Rn00561572_m1
Nos3	Nitric oxide synthase 3, endothelial cell	Rn02132634_s1
Npr1	Natriuretic peptide receptor A/guanylate cyclase A (atrionatriuretic peptide receptor A)	Rn00561678_m1
Npr2	Natriuretic peptide receptor B/guanylate cyclase B (atrionatriuretic peptide receptor B)	Rn00587693_m1
Npr3	Natriuretic peptide receptor C (atrionatriuretic peptide receptor C)	Rn00563495_m1
Pde1a	Phosphodiesterase 1A, calmodulin dependent	Rn01515459_m1
Pde2a	Phosphodiesterase 2A, cGMP stimulated	Rn00579346_m1
Pde3a	Phosphodiesterase 3A, cGMP inhibited	Rn00569192_m1
Pde5a	Phosphodiesterase 5A, cGMP specific	Rn01639345_m1
Ppia	Peptidylprolyl isomerase A (cyclophilin A)	Rn00690933_m1
Prkg1	Protein kinase, cGMP‐dependent, type 1	Rn01451055_m1
Prkg2	Protein kinase, cGMP‐dependent, type II	Rn00435938_m1
Slc9a3r1	Solute carrier family 9 (sodium/hydrogen exchanger), member 3 regulator 1	Rn00572154_m1

**Figure 7:**
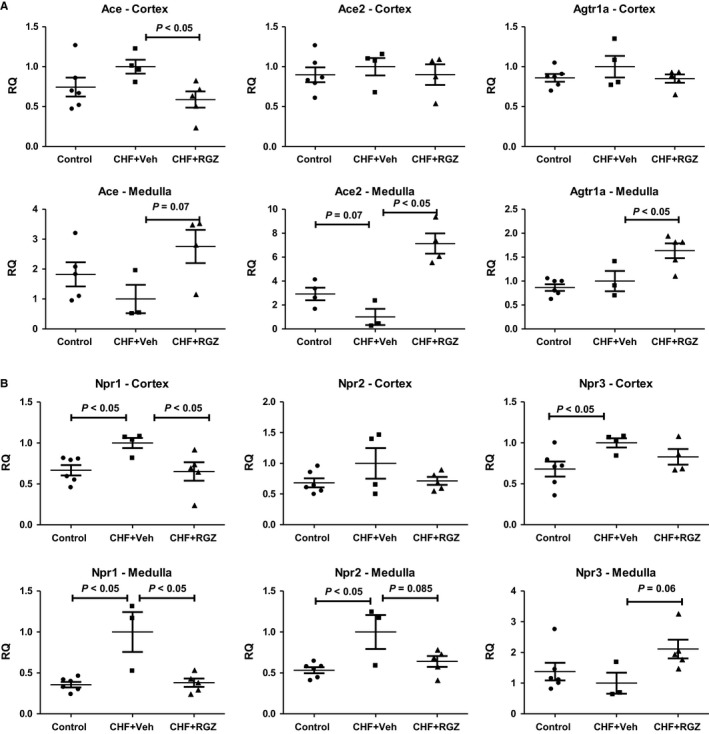
A, Effects of RGZ treatment on renal gene expression of RAAS components and Npr 2 in the renal cortex and medulla in CHF rats. RQ, relative quantification. B, Effects of 2‐wk RGZ treatment on renal gene expression of NP receptors in the renal cortex and medulla in CHF rats. RQ, relative quantification

**Figure 8:**
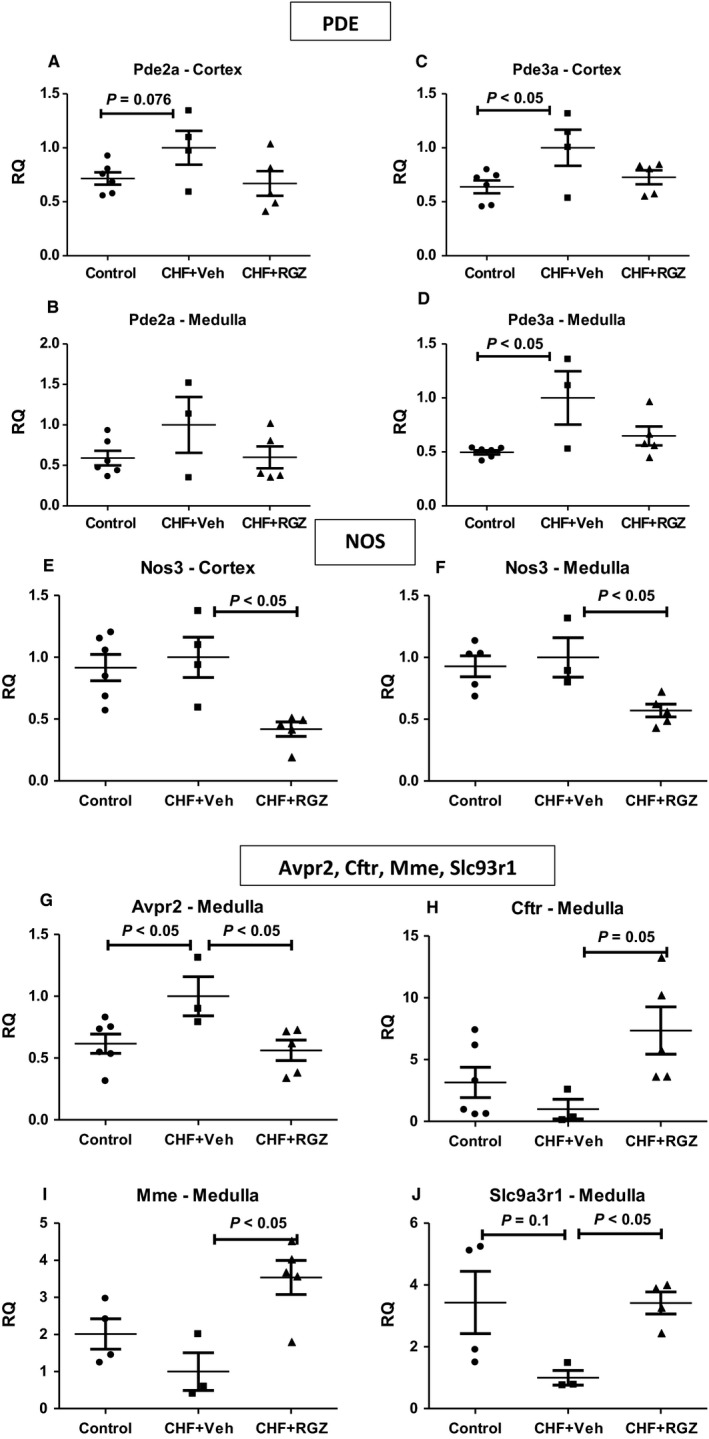
A, Effects of RGZ treatment on renal gene expression of selected PDE (A‐D), NOS isoforms (E‐F) and other selected genes (G‐J) in the renal cortex and medulla in CHF rats. RQ, relative quantification

When comparing renal expression of the tested genes between untreated CHF rats and control rats, it can be noticed that genes related to cGMP signalling (several PDE types but also NP receptors and sGC subunits) undergo up‐regulation in both renal cortices and medullae of CHF rats (Figures 7 and 8).

Based on these results, a validation study was performed to test the regulation of genes belonging to the following major categories: RAAS components (including the ACE2‐Ang1‐7‐Mas1 axis), NP signalling components (NPRs, PDEs, cGKs, metabolizing enzymes) and miscellaneous genes that include endothelial nitric oxide synthase (eNOS), NHE3 regulating factor 1 (NHERF1), V2R and CFTR (see Table 1).

Tables 2 and 3 summarize the significant alterations and trends in renal (cortical and inner medullary) expression of genes related to ANP signalling pathway. In addition, Figures 7 and 8 present the relative expression (denoted by relative quantification, RQ) of selected genes. After 2 weeks, the ‘CHF state’ was characterized by up‐regulation of several genes from the ANP signalling cascade, including NPRs1‐3, PDEs 2 and 3 and cGKI (denoted by Prkg1), varying in magnitude and trend towards statistical significance depending on the tissue examined. ACE2 and V2R (denoted by Avpr2) are down‐regulated and up‐regulated, respectively, in inner medullae of CHF rats compared with controls.

**Table 2:** Summary of ANP signalling‐related gene expression altered in the renal tissue of CHF rats compared with controls

Gene	RQ (fold expression)	P value
CHF vs Control—Cortex (each normalized to the housekeeper gene(s) of similar expression)		
Ace	1.34	0.15
Cftr	1.84	0.15
Npr1	1.5	**<0.05**
Npr3	1.47	**<0.05**
Pde2	1.4	0.076
Pde3	1.56	**<0.05**
Prkg1	1.47	0.1
CHF vs Control—Medulla (all normalized to an average of all housekeepers)		
Ace2	0.34	0.07
Avpr2	1.62	**<0.05**
Npr1	2.81	**<0.05**
Npr2	1.87	**<0.05**
Pde3	2.02	**<0.05**
Prkg1	1.5	**<0.05**
Slc9a3r1 (NHERF‐1)	0.29	0.1

Note

RQ, relative quantification.

Bold values represent statistical significance.

**Table 3:** Summary of ANP signalling‐related gene expression altered in the renal tissue of CHF rats treated with RGZ compared with the vehicle

Gene	RQ (fold expression)	P value
CHF+RGZ vs CHF+Veh—Cortex (each normalized to housekeeper gene of similar expression)		
Ace	0.58	**<0.05**
Nos3	0.42	**<0.05**
Npr1	0.65	**<0.05**
Pde2	0.67	0.13
Pde3	0.72	0.14
CHF+RGZ vs CHF+Veh—Medulla (all normalized to an average of all housekeepers)		
Ace	2.75	0.07
Ace2	6.07	**<0.05**
Agtr1a	1.63	**<0.05**
Avpr2	0.56	**<0.05**
Cftr	7.35	0.051
Mme	3.53	**<0.05**
Nos3	0.57	**<0.05**
Npr1	0.38	**<0.05**
Npr2	0.64	0.085
Npr3	2.11	0.06
Pde3	0.65	0.15
Prkg2	0.52	0.13
Slc9a3r1 (NHERF‐1)	2.98	**<0.05**

Note

RQ, relative quantification.

Bold values represent statistical significance.

Table 3 reports reduction of several RNA levels by RGZ treatment in CHF including NPR, PDE and cGK family (namely NPR1, PDEs 2 and 3 in the cortex and NPRs 1‐2, PDE3 and cGKII in the inner medulla), to levels similar to those in control rats. In this regard, the cortical expression of ACE, which was slightly but insignificantly increased in CHF rats vs controls, was down‐regulated in CHF+RGZ rats (0.58‐fold, *P* < 0.05) (Tables 2 and 3, Figure 7). The medullary expression of V2R was down‐regulated (0.56‐fold) and the expression of NHERF‐1 (denoted by Slc9a3r1, a gene which tended to decrease in CHF rats, threefold up‐regulation) was increased in response to RGZ treatment (*P* < 0.05 for both, Table 2 and 3, Figure 8).

Table 3 further shows that RGZ treatment reduced the cortical and medullary gene expression of eNOS (denoted by Nos3, 0.4‐ to 0.5‐fold, Figure 8) and significantly increased the medullary expression of CFTR (7.3‐fold, *P* = 0.05) and neutral endopeptidase (NEP, denoted by Mme, 3.5‐fold, *P* < 0.05).

When examining separately functionally clustered gene components such as RAAS components,^21^ in the renal medulla, RGZ treatment enhanced the expression of both ACE (2.75‐fold, *P* = 0.07) and ACE2 (sixfold, *P* < 0.05) although the degree of up‐regulation of the latter is more than twice larger. Also, the expression of AT1R is increased by RGZ treatment (1.6‐fold, *P* < 0.05). In addition, when inspecting the alterations in the NPR family genes (Figure 7), despite the general pattern of RGZ treatment in reducing back the expression of genes that were up‐regulated in CHF rats, the expression of NPR3 (coding for NPR‐C) in the renal medulla was up‐regulated about twofold (*P* < 0.05) independently of this pattern.

## 4 DISCUSSION

The present study extends our previous reports^21^ and provides novel information on the effects of prolonged RGZ treatment on renal haemodynamics and excretory functions in rats with ‘compensated’ CHF. Previously, we have demonstrated that chronic RGZ treatment caused no further increase in plasma volume compared with vehicle‐treated CHF rats, no increase in renal expression of Na^+^ transport‐linked channels/transporters, no association with any deterioration in selected biomarkers of CHF and did not result in worsening of the cardiac and renal status. The current study provides additional insights into the effects of RGZ on the renal response to various natriuretic and vasodilatory compounds including ANP, Ach and SNAP in rats with compensated CHF and assesses the impact of RGZ on gene expression related to the ANP signalling pathway. Specifically, CHF+RGZ rats exhibited significant enhancement in the natriuretic responses to ANP infusion, which are known to be blunted in CHF. However, GFR and the renal vasodilatory response to different vasodilators remained unaltered. This ‘sensitization’ to ANP was not associated with an increase in either urinary excretion of its second messenger cGMP or in vitro cGMP generation. RGZ‐regulated post‐cGMP signalling targets in CHF rats included several genes (ACE2, CFTR and V2R). In rats with ACF, RGZ did not exacerbate Na+ and water retention or cardiac dysfunction. Rather, it improved renal salt handling and ANP sensitivity, possibly through the enhancement of tubular post‐cGMP signalling.

Renal ‘resistance’ to the actions of ANP is a pathophysiological hallmark in CHF and involves perturbations in NPs metabolisms, effector generation and degradation along with physiological antagonism by neurohormonal effectors.^32^ In this regard, we found that RGZ treatment essentially restored the blunted renal excretory response to ANP infusion in CHF rats,^23^, ^27^ probably by ‘sensitizing’ the kidneys to ANP. This finding reflects the importance of the NPs system in maintaining salt and volume homoeostasis, determining prognosis and might be suggested as a therapeutic target in CHF.^13^

In support of our findings, Trivedi et al and others suggest that TZD treatment improves ANP‐induced natriuresis in insulin‐resistant rodents and patients without CHF, probably via increased ANP secretion and/or second messenger generation.^33^, ^34^, ^35^ Several publications reported that a week‐long treatment with RGZ in T2DM patients without clinical cardiovascular disease improved the natriuretic response to water immersion‐induced volume expansion, which is known to be blunted in diabetes. This improvement was accompanied by elevated plasma ANP levels and U_cGMP_V relative to untreated patients and was suggested to serve as an ‘escape’ mechanism from TZD‐induced fluid retention.^36^, ^37^, ^38^, ^39^ In our study, we also have found considerable improvements in the excretory responses to saline or ANP infusion in RGZ‐treated control rats, though to a lesser degree than in treated CHF rats. The improvement in the renal response to ANP in RGZ‐treated CHF rats is not related to circulating ANP levels, as these were the same as in CHF+Veh rats. Moreover, the obtained renal beneficial effects of RGZ were independent of enhanced generation of the second messenger cGMP in response to ANP, which was found largely unaltered both in vivo and in vitro, in CHF as well as in control rats. Thus, the findings so far suggest that RGZ improves the natriuretic response to ANP at a step beyond cGMP generation, namely at more downstream signalling events. This is in agreement with our previous study in our group that found similar cGMP‐producing capacity in rats with ACF‐induced CHF compared with sham controls.^27^

In order to further characterize the renal effects of RGZ, renal and cardiac responses to other manoeuvres such as acetylcholine and NO donors were also examined. In comparison with ANP employing particulate GC‐mediated signalling, Ach acts on the endothelium to generate NO, which in turn activates soluble GC.^40^ These two signalling systems are organized differently in terms of subcellular localization and mediate distinct physiological functions despite sharing the same second messenger.^41^ In this regard, some studies investigated renal NOS expression and urinary NO metabolite excretion as possible determinants of sodium handling in RGZ‐treated rats and found varying results according to the model or tissue type.^42^, ^43^, ^44^ At any rate, RGZ treatment did not significantly increase excretory response to ACh infusion in CHF rats, while some improvement was observed in control rats. This aligns with a previous work from our laboratory showing blunted ACh‐mediated renal vasodilatory response in rats with ACF,^25^ suggesting that RGZ selectively improves signalling transduced by particulate GC rather than soluble GC. In addition, to definitively exclude improved renal haemodynamics as the source for increased sodium excretion, we tested the effects of RGZ treatment on renal vasodilatation induced by ACh or SNAP. Since no difference was observed in RBF of RGZ‐treated CHF and control rats in response to either an endothelium‐dependent or an endothelium‐independent vasodilator, we concluded that the observed improvements in the renal handling of salt and water in CHF rats following RGZ treatment are not associated with alterations in renal haemodynamics. Furthermore, the lack of improvement in these renal haemodynamic responses supports the notion of selective improvement in particulate GC‐mediated signalling.

Since PPARγ acts as a transcription factor, we further looked for possible alterations in gene expression (Tables 1, 2, 3). When comparing renal expression of the tested genes between untreated CHF rats and control rats, it is noticeable that as a group, genes related to cGMP signalling tend to undergo up‐regulation in both renal cortices and medullae of CHF rats. When examining the alterations in gene expression in RGZ‐treated CHF rats compared with those treated with Veh, two general observations are apparent: (a) RGZ treatment reduced the expression of ANP signalling‐related genes that are up‐regulated in untreated CHF rats; (b) several individual genes of various physiological functions were differentially regulated by RGZ treatment regardless of their altered expression in the CHF disease state. On one hand, we found up‐regulation of genes coding to various PDE types (that degrade cGMP) and NPR‐C (which clears excess NPs), which may explain in part the tendency for sodium retention and blunted ANP‐induced natriuresis while displaying a similar cGMP‐producing capacity in our model.^27^ Although elevated renal activity of PDEs was shown previously in another model of tachypacing‐induced CHF, this is the first evidence of a similar notion in the ACF model.^45^, ^46^

As pointed out in the results, the RGZ‐induced alterations in gene expression seem to involve either down‐regulation of genes that were up‐regulated in CHF rats, and therefore perhaps implying a tendency towards ‘reversal’ of perturbed ANP signalling in CHF rats, or specific alterations not necessarily related to the changes observed in CHF state. In this regard, we found that RGZ‐treated CHF rats had reduced cortical ACE expression, in agreement with the finding in myocardial tissue and with another study in mesangial cells,^47^ and also in support of improved sodium handling in our study. Moreover, we found that RGZ treatment was associated with normalization of the increased inner medullary expression of V2R, in line with a previous study by our laboratory that found that antagonism of this receptor improved fluid retention and cardiac hypertrophy in our model.^48^

Some insights on the effects of RGZ on renal ANP signalling in CHF rats may be inferred from few genes that were found to be regulated in a distinctive manner by RGZ treatment (ie not reflecting normalization of alterations in CHF rats). For example, down‐regulation of renal eNOS expression may explain why we obtained no improvement in NO‐dependent renal vasodilatation in RGZ‐treated CHF rats. This stands in contrast with studies in RGZ‐treated rats without CHF which found increased protein abundance of eNOS.^42^, ^43^ Yet in line with the known impairment in renal vasodilatation in this model.^25^ We also found up‐regulation of the Mme gene, coding for NEP that degrades NPs. While this is contra intuitive, especially when considering the positive effects of NEP inhibition in CHF, it supports the notion that the improvement in ANP‐induced natriuresis in RGZ‐treated CHF rats is not related to ANP levels of cGMP generation.^22^

Two specific RGZ‐regulated genes in CHF rats may warrant special attention due to possible novel mechanistic insights regarding improved sodium handling in our model. The gene for CFTR, a major chloride‐secreting transporter in mammals, was found to be greatly up‐regulated (over sevenfold) by RGZ treatment in the medulla of CHF rats. Assuming increased expression of CFTR would be correlated with increased chloride secretion into the collecting duct lumen, resulting in increased luminal electronegativity that reduces the driving electrochemical gradient for sodium reabsorption in this tubular segment and known to be important for fine‐tuning renal sodium handling. The second potentially important finding is the up‐regulation of ACE2 relative to ACE1 (more than twofold) in the medulla of CHF+RGZ rats, despite both genes being up‐regulated by RGZ treatment. Considering the beneficial effects of the ACE2‐Ang1‐7‐Mas1 system and the counterregulatory role in opposing the classing RAS, this may provide another potential mechanism for the observed improvement in cardiac hypertrophy and sodium handling in RGZ‐treated CHF rats.^49^ In support of this notion of dual and perhaps interdependent regulation of both effectors, a previous study from our laboratory found increased cardiac ACE/ACE2 protein abundance and activity ratio in rats with ACF‐induced CHF.^50^

**Figure 9:**
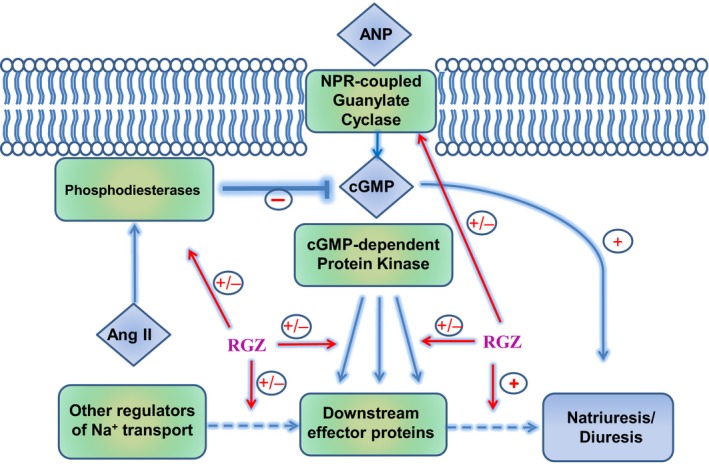
Schematic description of potential mechanisms underlying the beneficial effects of RGZ on kidney function. RGZ may affect the renal response to ANP at various levels including ANP, receptors, cGMP degradation or post cGMP downstream effector proteins. Briefly, RGZ was found to attenuate the up‐regulation of ACE, PDE and V2 receptor of AVP in the kidney, along enhancement of ACE2 expression. These effects may contribute to the beneficial effects of ANP on kidney function as was evident by enhance natriuresis and diuresis, either by post cGMP stage or at restoring the imbalance hormonal status/actions characterizing CHF

In summary, CHF+RGZ rats exhibited significant enhancement in the natriuretic responses to ANP infusion, which are known to be blunted in CHF. However, GFR and the renal vasodilatory response to different vasodilators remained unaltered. This ‘sensitization’ to ANP was not associated with increases in either urinary excretion of its second messenger cGMP or in vitro cGMP production. RGZ‐regulated post‐cGMP signalling targets in CHF rats included several genes (including ACE2, CFTR and V2 receptor) (Figure 9). Thus, the possibility that TZD treatment in uncomplicated CHF may be less detrimental than thought before deserves additional investigations. In particular, augmentation of cGMP‐mediated signalling is an attractive therapeutic strategy in CHF that is yet to be applied successfully in patients as a chronic treatment option.

## AUTHOR CONTRIBUTION

EG, JW and ZA designed the research study, performed the research, analysed the data and wrote the paper; EK, DA and ON analysed the data and wrote the paper; GZF designed the research study, provided essential compound (rosiglitazone) and wrote the paper.

## DISCLOSURE

The authors have no conflict of interest to disclose.
